# Oxidative/Antioxidative Status in Obese and Sport Trained Children: A Comparative Study

**DOI:** 10.1155/2015/315747

**Published:** 2015-03-31

**Authors:** Pawel Matusik, Zofia Prokopowicz, Berenika Norek, Magdalena Olszanecka-Glinianowicz, Jerzy Chudek, Ewa Malecka-Tendera

**Affiliations:** ^1^School of Medicine in Katowice, Department of Pediatrics, Pediatric Endocrinology and Diabetes, Medical University of Silesia, Medykow 16, 40-752 Katowice, Poland; ^2^School of Medicine in Katowice, Health Promotion and Obesity Management Unit, Department of Pathophysiology, Medical University of Silesia, Medykow 18, 40-752 Katowice, Poland; ^3^School of Medicine in Katowice, Pathophysiology Unit, Department of Pathophysiology, Medical University of Silesia, Medykow 18, 40-752 Katowice, Poland

## Abstract

The aim of the study was to compare oxidative/antioxidative status in obese and sport trained children and to correlate obtained redox markers with anthropometrical measurements, body composition parameters, and adipokines levels. 78 (44 males) obese (SG) and 80 (40 males) normal weight sport trained (CG) children matched for age and Tanner stage were recruited for the study. Body composition parameters and basal metabolic rate (BMR) were assessed by bioelectrical impedance analysis (BIA). Oxidative/antioxidative status was evaluated in plasma by total oxidative status (PerOX), oxidized-LDL cholesterol (oxLDL), total antioxidative capacity (ImAnOx), and glutathione peroxidase activity (GPx). Leptin and adiponectin levels and adiponectin/leptin ratio (A/L) were also investigated. OxLDL was higher in SG versus CG (*P* < 0.05), but ImAnOx and GPx were reduced in SG versus CG (*P* < 0.01). Redox markers correlated significantly with BMI *Z*-score, WHR, WHtR, body composition parameters, leptin (in boys only), and A/L ratio (in boys only) in SG and in a whole studied population. PerOX significantly correlated with BMR in the CG. Antioxidative/oxidative status in obese children is significantly impaired and related adipose tissue excess and its hormonal activity. Oxidative status assessed by PerOx is also high in sport trained children but antioxidative defense is significantly more efficient with no overproduction of oxidized LDL.

## 1. Introduction

Childhood obesity epidemic is nowadays the most important challenge for public health system worldwide, mainly in developed countries [1–4]. Childhood obesity is associated with increased risk of comorbidities as atherosclerosis and has an adverse impact on cardiovascular disease development in adulthood [5]. One of the important mechanisms of early atherosclerosis development is an oxidative stress, which occurs as a consequence of imbalance between the formation of reactive oxygen (ROS) and nitrogen species (RNS) and their inactivation by antioxidative defense system [6]. The most important step in this process is the generation of lipids peroxides as oxidized-LDL cholesterol (oxLDL). Adipose tissue is the major source of ROS production at the level of adipocyte mitochondria, during the processing of free fatty acids overload which can cause mitochondrial uncoupling and an increased release of incomplete electron transfer to oxygen [7, 8]. Recent studies showed that in obese children there is an important link between fat tissue-induced oxidative stress and adipokines production, inflammation, insulin resistance, and metabolic syndrome [8–13]. However, direct evidence coming from existing data based on small groups of patients, using different methods of oxidative stress assessment, is still limited. The most advisable treatment modality in childhood obesity is a lifestyle intervention based on a diet modification and increased physical activity [14]. On the other hand it is well known that exercise can promote the production of reactive species and free radicals itself [15]. Currently, data regarding exercise-induced oxidative stress in children and adolescent practicing sport are very limited [16–19]. Regular exercise training is associated with physiological adaptation of the body and consequently with numerous health benefits. On the other hand, exhausting and/or intense physical activity can induce diseases, injuries, and chronic fatigue, which can lead to the overtraining. There are just a few conflicting studies showing either lack or significant improvement in oxidative stress parameters after lifestyle intervention programs that included physical activity in obese children [20–22]. According to our knowledge, there are no comparative studies concerning oxidative/antioxidative status in obese and in practicing sport normal weight children. The aim of present study is to compare oxidative/antioxidative status in obese and sport trained children and to investigate the correlation of the redox markers with anthropometrical parameters, body composition, and adipokines levels.

## 2. Materials and Methods

### 2.1. Studied Population

Study group (SG) comprised 78 obese children in the mean age 13.96 ± 2.78 years. They were consecutively recruited for the study from the patients referred to our outpatients clinic at the Department of Pediatrics, Pediatric Endocrinology and Diabetes between September 2011 and March 2012. Children with syndromic obesity and endocrine disorders associated with obesity were excluded. Other exclusion criteria were factors that could influence oxidative status like infections, chronic diseases (i.e., asthma), and medications (i.e., antioxidant vitamins). None of the recruited obese children reported regular physical activity.

Control group (CG) comprised 82 team games players (girls training the handball and boys training soccer) recruited from the school sport clubs. Both boys and girls had very similar training schedule. They have been practicing at least three times per week with at least 1.5 h per session. They were all healthy, normal weight, and did not use neither medications nor diet supplements. They were asked not to engage in any heavy physical activity for 24 h before the blood sampling.

### 2.2. Anthropometric Measurements

Standing height was measured by a wall-mounted Harpender Stadiometer to the nearest 0.1 cm and weight (in patients in their underwear) by an electronic scale with readings accurate to 0.1 kg. Body mass index (BMI) was calculated, using the standard formula (kilograms per meter squared). BMI *Z*-scores were derived using WHO AnthroPlus, version 1.0.4 (based on World Health Organization growth references) [23]. Obesity was defined as BMI at or above the 95th percentile for age and sex, using the WHO charts [23]. Waist and hip circumferences were measured midway between the lower rib margin and the iliac crest in the standing position and waist hip ratio (WHR) and waist height ratio (WHtR) were calculated. For to the pubertal stage evaluation standard Tanner criteria were used [24].

### 2.3. Body Composition Analysis

Body composition parameters: fat mass (FAT), fat free mass (FFM), total body water (TBW), and predicted muscle mass (PMM) were assessed (in kilograms [kg] or as percentage of body weight [%]) based on bioelectrical impedance using segmental body composition analyzer (BC-418 MA Tanita Europe BV, Hoofddorp, Netherlands). Basal metabolic rate (BMR) in kJ/kg of body weight was evaluated by means of the same equipment.

### 2.4. Biochemical Analysis

Venous blood samples were drawn from antecubital vein in the morning in the supine position after the overnight fasting and collected in heparinized vacutainer tubes. After centrifugation at 1500 ×g at 4°C for 5 min, plasma was collected and transferred in Eppendorf tubes and then immediately frozen and stored at −80°C until analysis. Oxidative stress markers as total oxidative status/capacity (PerOX (TOS/TOC)), total antioxidative status/capacity (ImAnOx (TAS/TAC)), and glutathione peroxidase activity (GPx) were evaluated by using commercially available photometric technique (ImmunDiagnostik, Bensheim, Germany). Quantitative sandwich enzyme immunoassay technique was used for the measurement of oxidized low-density lipoprotein (oxLDL) (ImmunDiagnostik, Bensheim, Germany), leptin and adiponectin (TECOmedical AG, Swissach, Switzerland). Adiponectin/leptin ratio (A/L) was also calculated. All samples were tested in duplicate.

### 2.5. Statistical Analysis

The normal distribution of all the variables was confirmed by the Kolmogorov-Smirnov test. Baseline comparisons of categorical variables were performed by *χ* ^2^ test. Differences in continuous variables between studied groups were assessed by Student's *t*-test and adjusted for age, gender, and Tanner stage. Correlations between variables within each group were based on linear Pearson's correlation coefficient and were also adjusted for gender and Tanner stage. All statistical analyses were made by the Statistica 10 PL software and *P* < 0.05 was considered as significant. All results were reported as mean ± standard deviation (SD).

### 2.6. Ethics Approval

The study was approved by the Ethics Committee of Medical University of Silesia. All participants and/or their caregivers gave informed consent. Patient rights were also approved according to the Helsinki Declaration.

## 3. Results

### 3.1. Baseline Characteristics

Baseline characteristics and anthropometric measurements of all studied children are reported in Table 1. Subjects in the study and control groups were comparable with respect to age, gender, and Tanner stage distribution. As expected, there were strong significant differences between the groups concerning all anthropometrical variables and BMR.

### 3.2. Adipokines and Oxidative/Antioxidative Status

The adipokines levels and oxidative/antioxidative status of the study population are reported in Table 2. As expected, leptin level was significantly higher in the SG than in the CG (*P* < 0.0001) in either boys or girls. Adiponectin level was insignificantly lower in the SG, but a significant difference between studied groups was found in A/L ratio; however, the higher significance was noted for the boys (*P* < 0.001 versus *P* < 0.05, resp.). No significant difference was found in total oxidative stress (PerOX level) between SG and CG subjects. However lipid peroxidation expressed as OxLDL was significantly higher in the obese children compared to the sport trained children (*P* < 0.05). In addition, ImAnOx level and GPx activity were significantly reduced in SG compared to the CG (*P* < 0.01 for both parameters).

### 3.3. Correlation between Oxidative/Antioxidative Status and Nutritional Status and Adipokines Levels

All significant correlations found within the parameters in the study group are reported in Table 3. Significant correlations (adjusted for gender and Tanner stage) were shown for PerOX and BMI *Z*-score, WHtR, FAT%, and leptin (positive) versus FFM%, TBW%, and PMM% (negative). However leptin correlated significantly in boys only. Moreover, ImAnOx showed a significant negative correlation only with WHR. Oxidized-LDL (oxLDL) level and glutathione peroxidase activity (GPx) did not show any significant correlations in the SG.

However, in the total studied population (Table 4) we found significant positive correlations for the GPx with FFM%, TBW%, PMM%, and A/L (in boys only) and negative with BMI *Z*-score, WHR, WHtR, and FAT%. ImAnOx correlated significantly in the negative manner with BMI *Z*-score, WHR, and WHtR and positively with BMR/kg. PerOX level correlated significantly with body composition parameters and leptin level (in boys only) in the same manner as in the SG.

**Figure 1:**
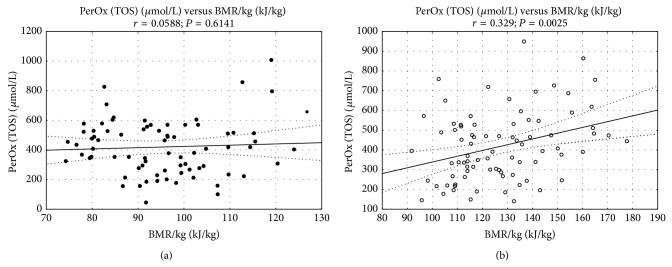
Correlation between total oxidative status (PerOX) and basal metabolic rate (BMR/kg) in the study (a) and control group (b).

Moreover, as depicted in Figure 1 there was a significant positive correlation between PerOX and BMR/kg in sport trained children.

## 4. Discussion

Increased generation of reactive oxygen species (ROS) in obesity has several backgrounds: dietary overload of macronutrients, mitochondrial dysfunction, excessive ROS generation on the level of endoplasmatic reticulum, and the inflammatory response [7, 8]. If there is no sufficient response from the system of antioxidative defense, oxidative stress develops.

Our study found that lipid peroxidation (oxLDL) was significantly stronger and antioxidative defense (ImAnOx and GPx) significantly weaker in obese than in sport trained children. Similar data was reported in the group of prepubertal obese children who had a significant decrease of glutathione peroxidase (GPx) and superoxide dismutase (SOD) activities [11]. Reduced total antioxidant status and lower *α*-tocopherol level in the group of obese children, particularly those with metabolic syndrome (MS), were reported by Molnár et al. [25]. In the recent study conducted by Faienza et al. [13], antioxidant/oxidant status was significantly altered in obese children with MS and no-MS compared to the lean controls.

In our study anthropometrical parameters (BMI *Z*-score, WHR, WHt/R), body composition values, and leptin level correlated significantly with oxidative/antioxidative status in the obese children as well as in the whole group. Furthermore, we found several important significant correlations between body composition parameters and oxidative/antioxidative status in both groups of children. Similar significant association between impaired oxidant/antioxidant status and BMI SDS in both obese and nonobese children was shown recently by Faienza et al. [13]. The importance of fat distribution in the context of oxidative stress in obesity was revealed by the other authors who found the correlation of anthropometrical parameters (WC and BMI *Z*-score) with level of oxidative stress activation [26, 27]. Visceral fat accumulation (measured by computed tomography), as a factor related to the enhanced oxidative status, has been also demonstrated by Araki et al. [28]. In the study by Kelly et al. [12], body fat accumulations measured by DXA, WC, and BMI were significantly related to the oxidized-LDL level. In our study, although oxLDL was significantly higher in the obese children, there was no significant relation between any adiposity parameters and its level. This may be due to the lipid profile disturbances occurring in the study group. Moreover, in our study the significant correlations between adiposity and both antioxidative (ImAnOx, GPx) and oxidative (PerOX) status have been demonstrated. Similar data concerning GPx are recently demonstrated by Spina et al. [29] in the adult population. They showed significant decrease of GPx activity in the subgroups of highest BMI category and the highest quartiles of WC.

Interestingly, total oxidative stress (PerOX) was at the same level in both groups. However, antioxidant defense was significantly stronger in the sport trained children. In fact, our results have documented that sport trained children, even if they have increased exercise-induced generation of ROS, activate expression of the components of the enzymatic antioxidative response as the adaptive process for the regular physical activity.

There is still a little data regarding exercise-induced oxidative stress, but study by Gomes et al. [15] showed a beneficial effect of the ROS produced during the regular physical activity (training adaptation, angiogenesis, mitochondria biogenesis, and muscle hypertrophy). In our study (in our knowledge based on the largest pediatric population) we demonstrated that oxidative/antioxidative status significantly depends on body composition and basal metabolic rate (BMR) in the lean sport trained children. Study by Djordjevic et al. [17] performed in the group of handball trained boys revealed that oxidative/antioxidative status is significantly related to their aerobic power (expressed as maximal oxygen uptake (VO_2_max)). Other studies were based on rather small groups of children [16, 30]. Importance of physical activity in reduction of oxidative stress markers in obese children was shown in several studies examining the lifestyle modification. Kelishadi et al. [21] reported significant reduction of oxidized-LDL and the other markers of oxidative stress after a short (6 weeks) lifestyle modification therapy, including aerobic physical activity. Regular physical activity can also lead to the increased expression of antioxidative enzymes, but this effect can be partially reversed in detraining [31]. However, Kelly et al. [22] observed no improvement of oxidative stress markers after 8 weeks of exercise training in the absence of weight reduction. Furthermore, in the absence of body fat reduction, there is no oxidative stress improvement with the physical activity alone and acute exercise challenge can even induce both inflammatory and oxidative stress markers elevation in obese children [32]. Particularly, an acute, unaccustomed, or eccentric exercise can induce muscle damage and may even decrease the efficiency of exercise therapy for metabolic improvement [33, 34]. It has to be also stated that generation of ROS, as well as their negative or positive impact, is closely related to the type of physical activity (aerobic versus anaerobic), its intensity and environment [35, 36]. Moreover, it may markedly influence the systemic effect of oxidative stress and obtained levels of antioxidative/oxidative stress markers. Significant increase of postexercise lipid peroxidation (ΔPerOX) in obese older women was found by Vincent et al. [37] after the maximal acute aerobic exercise. The same group found significant increase in both oxidative stress and antioxidative capacity levels in obese adult after acute resistance and aerobic exercise comparing to the nonobese controls. In both groups the exercise-induced changes in oxidative stress markers were associated with vitamin C intake, exercise ventilation rate, peak oxygen consumption (VO_2_peak), and plasma triglycerides. Moreover, exercise-induced lipid peroxides generation correlated significantly with body fat in the obese group [38]. Data showing the influence of acute exercise on oxidative/antioxidative balance in obese children are lacking. There is only little known about that phenomenon in the adolescent athletes. Nikolaidis et al. [18] showed significant increase in both oxidative and antioxidative markers in the adolescent swimmers after the 70–75% maximum velocity of acute swimming in a similar manner in boys and girls. However, antioxidative defense assessed as paraoxonase activity was significantly higher in the regular physically active adolescent athletes than in inactive control group of youth [19]. The positive results on antioxidative defense were recently assessed in soccer playing girls. There were significant increases of enzymatic activity (superoxide dismutase (SOD) and catalase (CAT)) while O_2_^−^ and H_2_O_2_ remained unchanged after the six months of regular training [39]. Therefore, further studies are needed regarding training type and intensity in obese children and adolescents. Based on the recent data, aerobic interval training may be the most useful also in the aspect of cardiovascular function improvement [40]. Moreover, proper antioxidants loads in the diet or their supplementation seem to have an additional beneficial effect on the exercise-induced oxidative stress [41].

In our study we found significant correlation between leptin level and oxidative stress (PerOX) in the group of boys both in obese and in the whole studied population, while adiponectin/leptin ratio correlated significantly with the GPx activity in boys from the whole studied group. Therefore, hormonal activity of adipose tissue seems to be interconnected with oxidative stress not only in obese children but also in lean physically active children and the boys are probably more afflicted. In the study conducted by Ustundag et al. [11] oxidative stress markers were strongly correlated with leptin level in prepubertal obese children. Therefore, oxidative stress vicious cycle can be perpetuated by inflammatory response in the very early stage of adiposity development. Dependency of oxidative stress level and inflammatory state on body fat mass was showed in the study of peripubertal children [32]. Thus, the key mechanism linking obesity with oxidative stress may be the impaired production of adipokines, provoked by the visceral adipose tissue accumulation.

The limitation of our study was the usage of bioelectrical impedance analysis (BIA) which is an indirect method of body composition assessment. However comparative studies have demonstrated a significant correlation between BIA data and body composition measured by densitometry (dual-energy X-ray absorptiometry (DXA)), which is the golden standard for that kind of analysis [42, 43]. Unfortunately, DXA is associated with exposure to X-ray radiation, making it a method of limited use in children. Moreover, BIA validation work among children and adolescents has resulted in the development of percentile charts for the healthy pediatric population [44]. Therefore, BIA seems to be a useful noninvasive tool for the body composition assessment in pediatric population. In particular, in our study body composition parameters correlated with oxidative/antioxidative status with the same manner as standard anthropometrical measures (BMI *Z*-score, WHR, and WHtR). Another parameter assessed indirectly based on BIA equipment was basal metabolic rate (BMR). However BMR assessment by direct calorimetry would have been too strenuous for children and very hard to perform in such a high number of volunteers. Oxidative stress is a multifaceted process and is difficult to quantify. That is why the other need is the usage of the other oxidative stress and oxidative damage parameters to raise the quality of final analysis and further conclusions. In our study the mixed (aerobic/anaerobic) training has been used as a control to the sedentary behavior in the obese children. However, direct measurements of the real exercise intensity will be certainly important. Therefore, further studies using daily monitoring of physical activity in both obese and sport trained children seem to be necessary.

## 5. Conclusions

In conclusion, our findings clearly demonstrate that oxidative/antioxidative balance is related to the body composition and fat tissue hormonal activity in obese children as well as in normal weight sport trained children and underline the importance of regular physical activity in the adaptation of antioxidative defense mechanisms. However, further studies are indicated to elucidate the role of physical activity induced oxidative stress in the behavioral intervention programs for obese children.

## Figures and Tables

**Table 1:** Baseline clinical characteristics and anthropometric measurements of studied groups.

	Study group (*n* = 78)	Control group (*n* = 82)	*P* value
Age (years)	13.96 ± 2.78	13.72 ± 2.4	NS
Sex (M : F)	44 : 34	40 : 42	NS
Height (cm)	163.51 ± 13.23	164.28 ± 12.05	NS
Tanner stages (I, II, III, IV)	23/12/26/12	26/16/29/13	NS
Weight (kg)	85.67 ± 21.60	49.13 ± 12.37	<0.0001
BMI *Z*-score (SD)	2.96 ± 1.16	0.38 ± 0.95	<0.0001
WHR	0.97 ± 0.07	0.81 ± 0.07	<0.0001
WHtR	0.62 ± 0.06	0.43 ± 0.04	<0.0001
FAT (%)	36.41 ± 7.36	21.84 ± 5.10	<0.0001
FFM (%)	63.59 ± 7.36	78.17 ± 5.09	<0.0001
PMM (%)	60.62 ± 7.09	76.07± 17.74	<0.0001
TBW (%)	46.56 ± 5.39	57.23 ± 3.73	<0.0001
BMR (kJ/kg)	95.59 ± 12.65	127.22 ± 19.66	<0.0001

Data are expressed as mean ± standard deviation.

BMI: body mass index, BMR: basal metabolic rate, FAT: fat mass, FFM: fat free mass, WHR; waist hip ratio, WHtR; waist height ratio, PMM: predicted muscle mass, TBW: total body water.

**Table 2:** Adipokines and oxidative/antioxidative status markers comparison of studied groups.

	Study group (*n* = 78)	Control group (*n* = 82)	*P* value
Leptin [ng/mL]			
Boys	23.9 ± 18.8	6.5 ± 3.4	<0.0001
Girls	28.3 ± 23.2	10.5 ± 5.9	<0.0001
Adiponectin [*μ*g/mL]	5.47 ± 4.41	6.16 ± 4.74	NS
Adiponectin/leptin (A/L)			
Boys	0.69 ± 1.51	5.01 ± 10.38	<0.001
Girls	0.33 ± 0.44	1.00 ± 1.57	<0.05
PerOX (TOS/TOC) [*μ*mol/L]	420.08 ± 182.93	418.41 ± 174.34	NS
ImAnOx (TAS/TAC) [*µ*mol/L]	133.30 ± 53.05	153.51 ± 28.97	<0.01
oxLDL [ng/mL]	608.79 ± 862.89	275.40 ± 215.63	<0.05
Glutathione peroxidase activity (GPx) [nmol/min/mL]	72.66 ± 24.59	83.98 ± 16.52	<0.01

Data are expressed as mean ± standard deviation.

PerOX (TOS/TOC): total oxidative status/capacity, ImAnOx (TAS/TAC): total antioxidative status/capacity, oxLDL: oxidized low-density lipoprotein.

**Table 3:** Significant correlations between nutritional status parameters, adipokines levels versus oxidative/antioxidative status markers in the study group.

Study group (*n* = 78)		

	ImOxAn (TAS/TAC) [*μ*mol/L]	
	Pearson's correlation	Significance

WHR	*r* = −0.272	*P* < 0.05

	PerOX (TOS/TOC) [*μ*mol/L]	
	Pearson's correlation	Significance

BMI *Z*-score (SD)	*r* = 0.245	*P* < 0.05
WHtR	*r* = 0.273	*P* < 0.05
FAT (%)	*r* = 0.423	*P* < 0.0001
FFM (%)	*r* = −0.423	*P* < 0.0001
TBW (%)	*r* = −0.423	*P* < 0.0001
PMM (%)	*r* = −0.419	*P* < 0.01
Leptin (ng/mL)		
Boys	*r* = 0.445	*P* < 0.01
Girls	*r* = −0.026	*P* = NS

BMI: body mass index, FAT: fat mass, FFM: fat free mass, ImAnOx (TAS/TAC): total antioxidative status/capacity, PerOX (TOS/TOC): total oxidative status/capacity, PMM: predicted muscle mass, TBW: total body water, WHR: waist hip ratio, WHtR: waist height ratio.

**Table 4:** Significant correlations between nutritional status parameters, adipokines levels versus oxidative/antioxidative status markers in total studied population.

Total (*n* = 160)		

	ImAnOx (TAS/TAC) [*μ*mol/L]	
	Pearson's correlation	Significance

BMI *Z*-score [SD]	*r* = −0.164	*P* < 0.05
WHR	*r* = −0.337	*P* < 0.001
WHtR	*r* = −0.305	*P* < 0.001
BMR/kg [kJ/kg]	*r* = 0.178	*P* < 0.05

	PerOX (TOS/TOC) [*μ*mol/L]	
	Pearson's correlation	Significance

FAT (%)	*r* = 0.218	*P* < 0.01
FFM (%)	*r* = −0.218	*P* < 0.01
TBW (%)	*r* = −0.218	*P* < 0.01
PMM (%)	*r* = −0.210	*P* < 0.01
Leptin [ng/mL]		
Boys	*r* = 0.252	*P* < 0.05
Girls	*r* = 0.088	*P* = NS

	Glutathione peroxidase activity (GPx)	
	[nmol/min/mL]	
	Pearson's correlation	Significance

BMI *Z*-score [SD]	*r* = −0.257	*P* < 0.01
WHR	*r* = −0.236	*P* < 0.05
WHtR	*r* = −0.309	*P* < 0.001
FAT (%)	*r* = −0.199	*P* < 0.05
FFM (%)	*r* = 0.200	*P* < 0.05
TBW (%)	*r* = 0.199	*P* < 0.05
PMM (%)	*r* = 0.202	*P* < 0.05
Adiponectin/leptin (A/L)		
Boys	*r* = 0.207	*P* < 0.05
Girls	*r* = −0.081	*P* = NS

BMI: body mass index, FAT: fat mass, FFM: fat free mass, ImAnOx (TAS/TAC): total antioxidative status/capacity, PerOX (TOS/TOC): total oxidative status/capacity, PMM: predicted muscle mass, WHR: waist hip ratio, WHtR: waist height ratio, TBW: total body water.

## References

[1] James P. T., Leach R., Kalamara E., Shayeghi M. (2001). The worldwide obesity epidemic. *Obesity Research*.

[2] Lobstein T., Frelut M.-L. (2003). Prevalence of overweight among children in Europe. *Obesity Reviews*.

[3] Małecka-Tendera E., Klimek K., Matusik P., Olszanecka-Glinianowicz M., Lehingue Y. (2005). Obesity and overweight prevalence in Polish 7to 9-year-old children. *Obesity Research*.

[4] Wang Y., Beydoun M. A. (2007). The obesity epidemic in the United States—gender, age, socioeconomic, racial/ethnic, and geographic characteristics: a systematic review and meta-regression analysis. *Epidemiologic Reviews*.

[5] Burke V. (2006). Obesity in childhood and cardiovascular risk. *Clinical and Experimental Pharmacology and Physiology*.

[6] Rice-Evans C., Burdon R. (1993). Free radical-lipid interactions and their pathological consequences. *Progress in Lipid Research*.

[7] Fujita K., Nishizawa H., Funahashi T., Shimomura I., Shimabukuro M. (2006). Systemic oxidative stress is associated with visceral fat accumulation and the metabolic syndrome. *Circulation Journal*.

[8] Codoñer-Franch P., Valls-Bellés V., Arilla-Codoñer A., Alonso-Iglesias E. (2011). Oxidant mechanisms in childhood obesity: the link between inflammation and oxidative stress. *Translational Research*.

[9] Chiavaroli V., Giannini C., D'Adamo E., de Giorgis T., Chiarelli F., Mohn A. (2009). Insulin resistance and oxidative stress in children born small and large for gestational age. *Pediatrics*.

[10] Codoñer-Franch P., Navarro-Ruiz A., Fernández-Ferri M., Arilla-Codoñer A., Ballester-Asensio E., Valls-Bellés V. (2012). A matter of fat: insulin resistance and oxidative stress. *Pediatric Diabetes*.

[11] Ustundag B., Gungor S., Aygün A. D., Turgut M., Yilmaz E. (2007). Oxidative status and serum leptin levels in obese prepubertal children. *Cell Biochemistry and Function*.

[12] Kelly A. S., Jacobs D. R., Sinaiko A. R., Moran A., Steffen L. M., Steinberger J. (2010). Relation of circulating oxidized LDL to obesity and insulin resistance in children. *Pediatric Diabetes*.

[13] Faienza M. F., Francavilla R., Goffredo R. (2012). Oxidative stress and metabolic syndrome in children and adolescents. *Hormon Research in Paediatrics*.

[14] Reinehr T. (2013). Lifestyle intervention in childhood obesity: changes and challenges. *Nature Reviews Endocrinology*.

[15] Gomes E. C., Silva A. N., de Oliveira M. R. (2012). Oxidants, antioxidants, and the beneficial roles of exercise-induced production of reactive species. *Oxidative Medicine and Cellular Longevity*.

[16] Gougoura S., Nikolaidis M. G., Kostaropoulos I. A., Jamurtas A. Z., Koukoulis G., Kouretas D. (2007). Increased oxidative stress indices in the blood of child swimmers. *European Journal of Applied Physiology*.

[17] Djordjevic D., Cubrilo D., MacUra M., Barudzic N., Djuric D., Jakovljevic V. (2011). The influence of training status on oxidative stress in young male handball players. *Molecular and Cellular Biochemistry*.

[18] Nikolaidis M. Q., Kyparos A., Hadziioannou M. (2007). Acute exercise markedly increases blood oxidative stress in boys and girls. *Applied Physiology, Nutrition and Metabolism*.

[19] Cakmak A., Zeyrek D., Atas A., Erel O. (2010). Paraoxonase activity in athletic adolescents. *Pediatric Exercise Science*.

[20] Mohn A., Catino M., Capanna R., Giannini C., Marcovecchio M., Chiarelli F. (2005). Increased oxidative stress in prepubertal severely obese children: effect of a dietary restriction-weight loss program. *Journal of Clinical Endocrinology and Metabolism*.

[21] Kelishadi R., Hashemi M., Mohammadifard N., Asgary S., Khavarian N. (2008). Association of changes in oxidative and proinflammatory states with changes in vascular function after a lifestyle modification trial among obese children. *Clinical Chemistry*.

[22] Kelly A. S., Steinberger J., Olson T. P., Dengel D. R. (2007). In the absence of weight loss, exercise training does not improve adipokines or oxidative stress in overweight children. *Metabolism*.

[23] de Onis M., Onyango A. W., Borghi E., Siyam A., Nishida C., Siekmann J. (2007). Development of a WHO growth reference for school-aged children and adolescents. *Bulletin of the World Health Organization*.

[24] Tanner J. M., Whitehouse R. H. (1976). Clinical longitudinal standards for height, weight, height velocity, weight velocity, and stages of puberty. *Archives of Disease in Childhood*.

[25] Molnár D., Decsi T., Koletzko B. (2004). Reduced antioxidant status in obese children with multimetabolic syndrome. *International Journal of Obesity*.

[26] Kelishadi R., Sharifi M., Khosravi A., Adeli K. (2007). Relationship between C-reactive protein and atherosclerotic risk factors and oxidative stress markers among young persons 10–18 years old. *Clinical Chemistry*.

[27] Codoner-Franch P., Boix-Garcia L., Simo-Jorda R., Castillo-Villaescusa C., Maset-Maldonado J., Valls-Belles V. (2010). Is obesity associated with oxidative stress in children?. *International Journal of Pediatric Obesity*.

[28] Araki S., Dobashi K., Yamamoto Y., Asayama K., Kusuhara K. (2010). Increased plasma isoprostane is associated with visceral fat, high molecular weight adiponectin, and metabolic complications in obese children. *European Journal of Pediatrics*.

[29] Spina A., Guallar E., Rayman M. P., Tigbe W., Kandala N.-B., Stranges S. (2013). Anthropometric indices and selenium status in British adults: the U.K. National Diet and Nutrition Survey. *Free Radical Biology and Medicine*.

[30] Nasca M. M., Zhang R., Super D. M., Hazen S. L., Hall H. R. (2010). Increased oxidative stress in healthy children following an exercise program: a pilot study. *Journal of Developmental and Behavioral Pediatrics*.

[31] Woo J., Shin K. O., Yoo J.-H., Park S., Kang S. (2012). The effects of detraining on blood adipokines and antioxidant enzyme in Korean overweight children. *European Journal of Pediatrics*.

[32] Oliver S. R., Rosa J. S., Milne G. L. (2010). Increased oxidative stress and altered substrate metabolism in obese children. *International Journal of Pediatric Obesity*.

[33] Aoi W., Naito Y., Yoshikawa T. (2013). Role of oxidative stress in impaired insulin signaling associated with exercise-induced muscle damage. *Free Radical Biology and Medicine*.

[34] Aoi W., Naito Y., Takanami Y. (2004). Oxidative stress and delayed-onset muscle damage after exercise. *Free Radical Biology and Medicine*.

[35] Margonis K., Fatouros I. G., Jamurtas A. Z. (2007). Oxidative stress biomarkers responses to physical overtraining: implications for diagnosis. *Free Radical Biology and Medicine*.

[36] Knez W. L., Periard J. P. (2014). The impact of match-play tennis in a hot environment on indirect markers of oxidative stress and antioxidant status. *British Journal of Sports Medicine*.

[37] Vincent H. K., Vincent K. R., Bourguignon C., Braith R. W. (2005). Obesity and postexercise oxidative stress in older women. *Medicine and Science in Sports and Exercise*.

[38] Vincent H. K., Morgan J. W., Vincent K. R. (2004). Obesity exacerbates oxidative stress levels after acute exercise. *Medicine and Science in Sports and Exercise*.

[39] Zivkovic V., Lazarevic P., Djuric D. (2013). Alteration in basal redox state of young male soccer players after a six-month training programme. *Acta Physiologica Hungarica*.

[40] Ingul C. B., Tjonna A. E., Stolen T. O., Stoylen A., Wisloff U. (2010). Impaired cardiac function among obese adolescents: effect of aerobic interval training. *Archives of Pediatrics and Adolescent Medicine*.

[41] Vincent H. K., Bourguignon C. M., Vincent K. R., Weltman A. L., Bryant M., Taylor A. G. (2006). Antioxidant supplementation lowers exercise-induced oxidative stress in young overweight adults. *Obesity*.

[42] de Lorenzo A., Sorge S. P., Iacopino L., Andreoli A., de Luca P. P., Sasso G. F. (1998). Fat-free mass by bioelectrical impedance vs dual-energy X-ray absorptiometry (DXA). *Applied Radiation and Isotopes*.

[43] Thomson R., Brinkworth G. D., Buckley J. D., Noakes M., Clifton P. M. (2007). Good agreement between bioelectrical impedance and dual-energy X-ray absorptiometry for estimating changes in body composition during weight loss in overweight young women. *Clinical Nutrition*.

[44] McCarthy H. D., Cole T. J., Fry T., Jebb S. A., Prentice A. M. (2006). Body fat reference curves for children. *International Journal of Obesity*.

